# Electrical Impedance Tomography (EIT) in a Patient Suffering from Post-COVID Syndrome with Dyspnea: A Case Report

**DOI:** 10.3390/diagnostics12102284

**Published:** 2022-09-21

**Authors:** Katrin Katzer, Yvonne Gremme, Majd Moshmosh Alsabbagh, Andreas Stallmach, Philipp Reuken, Jan-Christoph Lewejohann

**Affiliations:** 1 Department of Internal Medicine IV, Jena University Hospital, Friedrich-Schiller-University, 07747 Jena, Germany; 2 Center for Sepsis Control and Care (CSCC), Jena University Hospital, Friedrich-Schiller-University, 07747 Jena, Germany; 3 Department of Emergency Medicine, Jena University Hospital, Friedrich-Schiller-University, 07747 Jena, Germany

**Keywords:** electrical impedance tomography (EIT), post-COVID syndrome, distribution of ventilation

## Abstract

Background: Long-term health consequences following COVID-19 disease constitute an increasing problem worldwide. A considerable number of patients still suffer from various symptoms, most commonly dyspnea, months or even years after the acute infection. In these patients, a classical pulmonary function test often yields no significant findings. Subsequently, treating those patients is a challenge for any physician as there are currently no evidence-based treatment plans. Case and methods: We reported the case of a 58-year-old patient who was still suffering from resting dyspnea six months after severe COVID-19 pneumonia. The dyspnea was so pronounced that the patient was supplied with home oxygen, which they used as needed. The regional distribution of ventilation in the lungs was studied twice utilizing noninvasive electrical impedance tomography (EIT). The first examination showed distinct inhomogeneities of regional ventilation, a regional ventilation delay (RVD) of 15%, and pronounced pendelluft phenomena. Seven weeks after treatment with budesonide and physical therapy, the patient reported a clear subjective improvement in complaints. Accordingly, the regional distribution of ventilation also improved. Conclusion: Electrical impedance tomography might be a promising method to assess lung function in post-COVID patients; however, controlled and larger studies are necessary.

## 1. Background

Post-COVID syndrome is a multiorgan disease with currently unknown prevalence. As clear definitions of post-COVID syndrome have only been developed in recent months and probably a high number of patients still suffers from various symptoms without ever receiving the correct diagnosis, it is still unclear how many patients develop this long-term sequela after acute SARS-CoV-2 infection. According to various estimates, the figure ranges from 2.5% according to individual reports up to 72.5% as reported in a systematic review with a large selection bias to be assumed [1]. Even if the prevalence is low, the importance for health and social systems is considerable due to the high number of SARS-CoV-2 infections worldwide. The pathogenesis remains unclear. Patients with post-COVID syndrome often suffer from shortness of breath, fatigue, as well as reduced physical and mental performance for months or even years after acute SARS-CoV-2 infection [2,3,4]. 

In patients with dyspnea, conventional diagnostic procedures (computer tomography, spirometry, body plethysmography, evaluation of diffusion capacitance) in many cases do not show pathological changes, making it difficult for the treating physician to choose the appropriate therapeutic approach [5] and monitor therapeutic effects [6,7,8,9]. There are no evidence-based treatment plans for patients suffering from dyspnea with inconspicuous results before the mentioned conventional diagnostic procedures; e.g., the German guidelines do not recommend routine antifibrotic treatment in all patients suffering from dyspnea but only if an imaging procedure shows signs of fibrosis.

## 2. The Case and the Method

We report a 58-year-old patient who first presented to our post-COVID outpatient clinic roughly six months after severe COVID-19 pneumonia. Shortly before SARS-CoV-2 infection, the causative meningioma was resected after the first seizure. Postoperatively, the patient suffered from pulmonary embolism, which was treated with an anticoagulant (Apixaban). A few days after discharge from the hospital, the patient developed flu-like symptoms with progressive cough. A PCR test showed acute SARS-CoV-2 infection as the cause. Initial computed tomography of the chest showed bipulmonary infiltrates, typically observed in patients with COVID-19 pneumonia [10]. Because of severe respiratory insufficiency, the patient had to be intubated and mechanically ventilated for about five weeks at an external hospital. Consequently, a tracheotomy was performed during the patient’s stay in the ICU. 

Complications included tension pneumothorax on the left side and acute delirium. After treatment at the intensive care unit, the patient was transferred directly to a rehabilitation clinic. Weaning of ventilation was successful, and the tracheostomy could be removed during the stay at the rehabilitation clinic.

On the first presentation to our post-COVID outpatient clinic, the obese patient (BMI of 39.8 kg/m²) with hypertensive blood pressure (RR of 159/116 mmHg) and marked resting dyspnea (SpO_2_ of 85–90%) reported significant resting and exertional dyspnea together with intermittent oxygen demand, impaired concentration, fatigue, and symptoms of critical illness, polyneuropathy, and myopathy.

Auscultation of the lungs and heart was normal. ECG and transthoracic echocardiography ruled out a cardiac disease as the cause of the complaints. The laboratory values were normal. 

Computed tomography showed ground-glass opacities in both lungs, consistent with changes after severe COVID-19 pneumonia [11]. There were no signs of structural changes in the pulmonary framework such as pulmonary fibrosis, pulmonary emphysema, or scars [12]. Pulmonary function tests revealed signs of restrictive ventilation disorder and a slightly reduced diffusion capacity. FEV1 and FVC were significantly reduced, with 2.65 L (67.5% of the expected value) and 3.19 L (62.5%), respectively. The Tiffeneau index was normal (83%). DLCO was also reduced (8.09 mmol/(min*kPa), 76.4% of the expected value) and the transfer coefficient (Krogh index) was normal (1.72). The arterial blood gas analysis showed normal values (pH 7.42; paO_2_, 75 mm Hg; paCO_2_, 36 mm Hg; bicarbonate, 24 mmol/L). In the 6-min walk test, the patient achieved a walking distance of 360 m (55% of the expected distance). 

**Figure 1:**
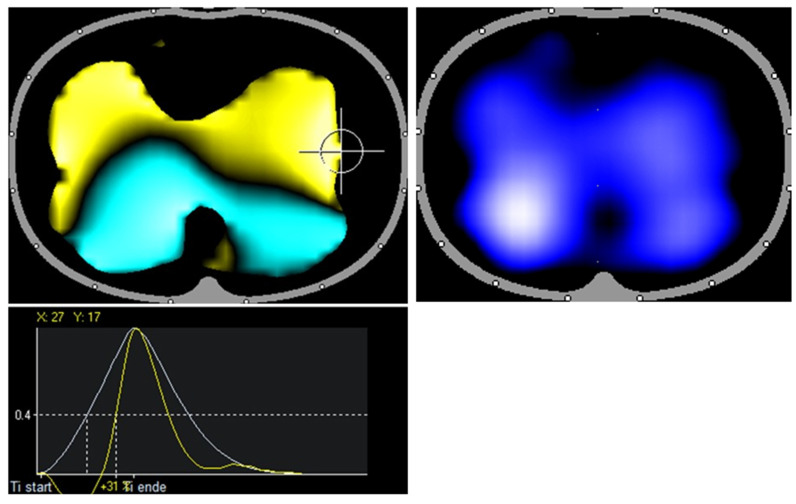
Healthy volunteer: RVD image shows symmetrical ventilation to both lungs up to the marginal areas. The air first reaches the dorsal lung regions symmetrically on both sides (turquoise area) and the ventral lung regions (yellow area). Note especially the lateral symmetry and the calm distribution of the colors.

To further check the lung function ventilation, we performed chest electrical impedance tomography (EIT) to visualize regional lung ventilation. With the help of EIT, impedance changes of the lungs, which change with the air content in healthy lungs by a factor of 300, can be measured via electrodes on the body surface (Figure 1) [10]. 

Since EIT visualizes regional ventilation distribution, it could have an additional diagnostic value in the assessment of dyspnea in the context of the post-COVID syndrome. It is a radiation-free noninvasive imaging technique that can be used at the bedside and has been used for many years in intensive care units to monitor ventilated patients; therefore, little is known about EIT imaging in other patients. For EIT measurement, an electrode belt with 16 surface electrodes is placed around the thorax at the level of the 4th intercostal space and a reference electrode is placed on the abdomen. The patient is in the supine position and breathing spontaneously. Measurement of the distribution of intrathoracic bioimpedance is performed by applying a known very low alternating current to the first pair of electrodes and measuring the resulting voltage changes of less than 100 μV at the remaining 13 pairs of electrodes. The position of the injecting and measuring electrode pairs rotates successively around the entire thorax so that a high temporal resolution is achieved. One complete rotation produces voltage profiles at 16 electrode positions, each consisting of 13 voltage measurements. The resulting 208 values or frames are used to reconstruct an EIT cross-sectional image. The 208 frames are converted into an ellipsoidal EIT image using a linearized Newton–Raphson reconstruction algorithm based on the finite element method [11]. In spontaneously breathing patients, EIT has been used sporadically in clinical trials, and no reference values are available [12]. Clear advantages of EIT include mobility, bedside detection of regional ventilatory disturbances in near real-time, high sensitivity, repeatability, and freedom from radiation. The validity and reproducibility of EIT have been demonstrated in several experimental and clinical studies using different reference methods such as CT, SPECT, and PET. EIT shows a high correlation with lung air content compared with computed tomography [13]. In critically ill patients, regional ventilation shows good agreement between EIT and dynamic CT [14]. Comparison of EIT with ventilation scintigraphy shows good accordance [15,16]. A comparison of increased lung volume during inspiration shows good agreement between plethysmography and impedance changes in EIT [17].

We used an EIT device from Dräger Medical, Lübeck, Germany. For the analysis, the artefact filter and the low-pass filter of 50 min were switched on. The manufacturer’s software (version 1.30) was used for the analysis. 

The use of the data was approved by the local ethics committee (2020-1978-Daten) and the work was conducted in accordance with the declaration of Helsinki on good clinical practice, and the patient provided written informed consent for the publication of their personal data.

**Figure 2:**
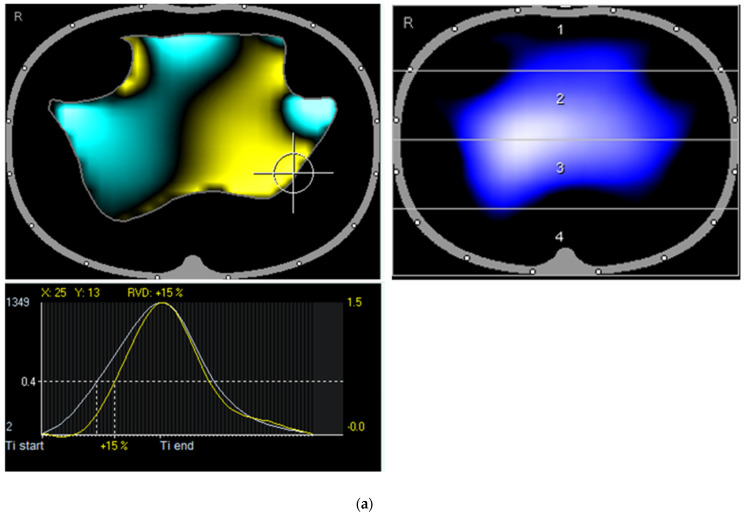

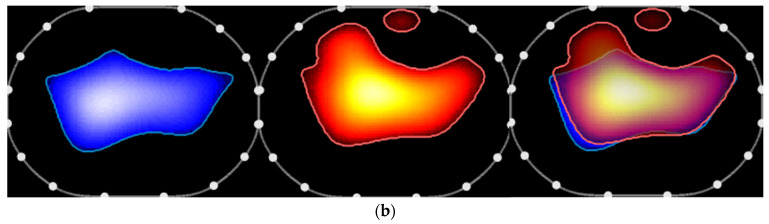
(**a**) First presentation of the patient: Tidal image shows reduced ventilation on both ventral sides and dorsal left. The enhanced color map shows a visually recognizable irregular shape of the ventilation contour similar to thorn apple forms. Recognizable is a pronounced regional ventilation delay (left dorsal; +15 %). (**b**) First presentation of the patient: Largely consistent distribution of ventilation and pulsatile blood flow, except for a small right ventral right region, where pulsation but no ventilation is observed.

The first examination showed reduced ventilation in the dorsal and ventral left regions in the tidal image, while the extended color map showed a visually recognizable irregular shape of the ventilation. In addition, there was a pronounced left dorsal regional ventilation delay (RVD) as an expression of a significantly delayed inspiratory and slightly premature expiratory course of regional ventilation in this region compared to the right dorsal region (Figure 2a). Furthermore, typical patterns of cyclical opening and closing of the dorsally located alveoli in the transition between collapsed and ventilated areas with a late opening were observed. The pronounced pendelluft phenomena, defined as asynchronous alveolar ventilation, were obvious [18]. After the pulsatility analysis (circulation-related impedance change), the distribution of ventilation and pulsatility was highly concordant, except for a small right ventral region, where pulsation (but no ventilation) was observed, presumably only due to scaling effects (Figure 2b).

The patient was then treated with budesonide inhalations twice daily and intensive physical therapy with an emphasis on breathing techniques. The oral medication was left unchanged.

**Figure 3:**
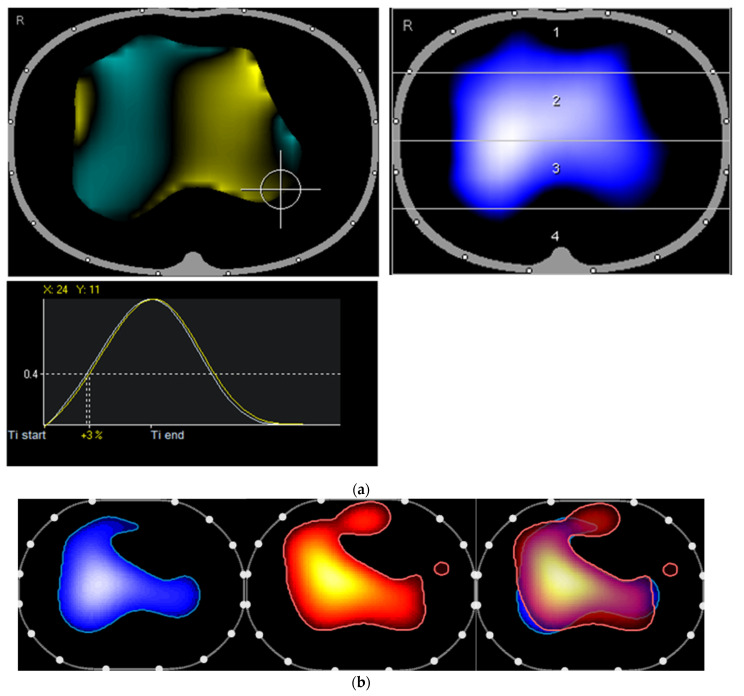
(**a**) The second presentation of the patient: The ventilated area is at this time slightly increased with continued low ventilation (left ventral in the tidal image). The inspiratory course is more homogeneous, with only a small extent of the regional ventilation delay (left dorsal; +3%) and a lateroventrally located region that is ventilated earlier (turquoise). (**b**) Second presentation of the patient: The right ventral region is now ventilated again. The distribution of ventilation and pulsatile blood flow are highly concordant.

At the follow-up visit to our post-COVID outpatient clinic seven weeks later, the patient presented in a better general condition, reporting better physical performance and decreased dyspnea. The oxygen was no longer needed. We performed a repeat EIT, which revealed a slightly increased ventilated area and continued low ventilation left ventral in the tidal image. The inspiratory course was much more homogeneous in this second measurement (Figure 3a). In the pulsatility analysis, the distribution of ventilation and pulsatility was highly consistent, except for a small right ventral region where pulsation but no ventilation was observed (Figure 3b).

Both drug and physical therapy were continued. The next step in treatment is an inpatient rehabilitation program that focuses on improving the breathing and cognitive functions.

## 3. Discussion

In our case report, we show a distinct impairment of regional ventilation distribution with pendelluft phenomena utilizing EIT in a patient suffering from marked resting and exertional dyspnea six months after SARS-CoV-2 infection. To our knowledge, this was the first time such an investigation has been performed in a in a spontaneously breathing post-Covid syndrome patient suffering from resting and exertional dyspnea and the first time that regional ventilation impairment has been demonstrated following acute SARS-CoV-2 infection. EIT is the first examination method that visualizes these phenomena without radiation from the bedside and offers the possibility to assess the effects of different treatment strategies in post-COVID patients. This is a significant step in improving the care of post-COVID patients who often suffer from dyspnea without having any noticeable problems or pathological findings in conventional diagnostic procedures. 

Furthermore, we were able to show that after therapy with budesonide inhalations and comprehensive physical therapy with a focus on breathing techniques, both the regional ventilation distribution and the homogeneity of inspiratory dynamics presented by RVD could be visualized by EIT, and the patient’s subjectively described symptoms improved. This is another significant finding, as there are no evidence-based treatment plans for patients suffering from post-COVID syndrome with dyspnea. In our patient, combined treatment of budesonide inhalations and comprehensive physical therapy enabled our patient to be independent of oxygen insufflation once more and improved their overall well-being.

However, our patient not only had severe COVID-19 pneumonia, but also previous pulmonary embolism. In our estimation, the changes in EIT were not a consequence of pulmonary embolism that had occurred before the SARS-CoV-2 infection as the patient was receiving anticoagulation therapy and no residual emboli were detectable on computed tomography.

EIT is a noninvasive examination technique that has mainly been used for monitoring mechanically ventilated patients [19]. Our results show that it also provides valuable information in spontaneously breathing patients. In contrast to CT, EIT can visualize lung function over time and present region-specific information about the distribution of ventilation in the lungs and is a radiation-free imaging technique, so it can be used in younger patients and for repeated examinations. Furthermore, experience with patients suffering from persistent dyspnea after SARS-CoV-2 infection often shows no impairment in lung function and computed tomography [5]. Because of the limited experience with EIT in spontaneously breathing patients with post-COVID syndrome, it would be desirable to combine EIT with pulmonary function tests (PFT) [20], which are the gold standard, and cardiopulmonary exercise testing [21] on the same day.

We conclude that EIT is a suitable examination procedure for visualization of regional changes in ventilation distribution in patients with post-COVID syndrome. If these are detected by EIT, we recommend intensive physical therapy with a focus on breathing techniques as well as regular inhalation treatment.

## 4. Limitations

As this is only a case report, our findings are limited to one patient. Controlled and larger studies are needed to validate the EIT as a proven examination procedure in patients with post-COVID suffering from dyspnea. Additional limitations arise from the fact that the patient was not treated in our clinic during their acute COVID pneumonia. Therefore, we do not know all the details about their treatment during the infection as well as during their rehabilitation. 

## Data Availability

The data presented in this study are available on request from the corresponding author.

## References

[1] Nasserie T., Hittle M., Goodman S.N. (2021). Assessment of the Frequency and Variety of Persistent Symptoms Among Patients With COVID-19: A Systematic Review. JAMA Netw. Open.

[2] Groff D., Sun A., Ssentongo A.E., Ba D.M., Parsons N., Poudel G.R., Lekoubou A., Oh J.S., Ericson J.E., Ssentongo P. (2021). Short-term and Long-term Rates of Postacute Sequelae of SARS-CoV-2 Infection: A Systematic Review. JAMA Netw. Open.

[3] Nalbandian A., Sehgal K., Gupta A., Madhavan M.V., McGroder C., Stevens J.S., Cook J.R., Nordvig A.S., Shalev D., Sehrawat T.S. (2021). Post-acute COVID-19 syndrome. Nat. Med..

[4] Stallmach A., Kesselmeier M., Bauer M., Gramlich J., Finke K., Fischer A., Fleischmann-Struzek C., Heutelbeck A., Katzer K., Mutschke S. (2022). Comparison of fatigue, cognitive dysfunction and psychological disorders in post-COVID patients and patients after sepsis: Is there a specific constellation?. Infection.

[5] Solomon J.J., Heyman B., Ko J.P., Condos R., Lynch D.A. (2021). CT of Post-Acute Lung Complications of COVID-19. Radiology.

[6] Guler S.A., Ebner L., Aubry-Beigelman C., Bridevaux P.-O., Brutsche M., Clarenbach C., Garzoni C., Geiser T.K., Lenoir A., Mancinetti M. (2021). Pulmonary function and radiological features 4 months after COVID-19: First results from the national prospective observational Swiss COVID-19 lung study. Eur. Respir. J..

[7] Sonnweber T., Sahanic S., Pizzini A., Luger A., Schwabl C., Sonnweber B., Kurz K., Koppelstätter S., Haschka D., Petzer V. (2021). Cardiopulmonary recovery after COVID-19: An observational prospective multicentre trial. Eur. Respir. J..

[8] Komici K., Bianco A., Perrotta F., Iacono A.D., Bencivenga L., D’Agnano V., Rocca A., Bianco A., Rengo G., Guerra G. (2021). Clinical Characteristics, Exercise Capacity and Pulmonary Function in Post-COVID-19 Competitive Athletes. J. Clin. Med..

[9] Joris M., Minguet P., Colson C., Joris J., Fadeur M., Minguet G., Guiot J., Misset B., Rousseau A.-F. (2021). Cardiopulmonary Exercise Testing in Critically Ill Coronavirus Disease 2019 Survivors: Evidence of a Sustained Exercise Intolerance and Hypermetabolism. Crit. Care Explor..

[10] Churruca M., Martinez-Besteiro E., Counago F., Landete P. (2021). COVID-19 pneumonia: A review of typical radiological characteristics. World J. Radiol..

[11] Manolescu D., Timar B., Bratosin F., Rosca O., Citu C., Oancea C. (2022). Predictors for COVID-19 Complete Remission with HRCT Pattern Evolution: A Monocentric, Prospective Study. Diagnostics.

[12] Orlandi M., Landini N., Sambataro G., Nardi C., Tofani L., Bruni C., Randone S.B., Blagojevic J., Melchiorre D., Hughes M. (2022). The role of chest CT in deciphering interstitial lung involvement: Systemic sclerosis versus COVID-19. Rheumatology.

[13] Frerichs I., Hinz J., Herrmann P., Weisser G., Hahn G., Dudykevych T., Quintel M., Hellige G. (2002). Detection of local lung air content by electrical impedance tomography compared with electron beam CT. J. Appl. Physiol..

[14] Victorino J.A., Borges J.B., Okamoto V.N., Matos G.F., Tucci M.R., Caramez M.P., Tanaka H., Sipmann F.S., Santos D.C.B., Barbas C.S.V. (2004). Imbalances in regional lung ventilation: A validation study on electrical impedance tomography. Am. J. Respir. Crit. Care Med..

[15] Kunst P.W., Noordegraaf A.V., Hoekstra O.S., Postmus P.E., De Vries P.M. (1998). Ventilation and perfusion imaging by electrical impedance tomography: A comparison with radionuclide scanning. Physiol. Meas..

[16] Serrano R.E., de Lema B., Casas O., Feixas T., Calaf N., Camacho V., Carrió I., Casan P., Sanchis J., Riu P.J. (2002). Use of electrical impedance tomography (EIT) for the assessment of unilateral pulmonary function. Physiol. Meas..

[17] Marquis F., Coulombe N., Costa R., Gagnon H., Guardo R., Skrobik Y. (2006). Electrical impedance tomography’s correlation to lung volume is not influenced by anthropometric parameters. J. Clin. Monit. Comput..

[18] Sang L., Zhao Z., Yun P.-J., Frerichs I., Möller K., Fu F., Liu X., Zhong N., Li Y. (2020). Qualitative and quantitative assessment of pendelluft: A simple method based on electrical impedance tomography. Ann. Transl. Med..

[19] Sang L., Zhao Z., Lin Z., Liu X., Zhong N., Li Y. (2020). A narrative review of electrical impedance tomography in lung diseases with flow limitation and hyperinflation: Methodologies and applications. Ann. Transl. Med..

[20] Huntley C.C., Patel K., Bil Bushra S.E., Mobeen F., Armitage M.N., Pye A., Knight C.B., Mostafa A., Kershaw M., Mughal A.Z. (2022). Pulmonary function test and computed tomography features during follow-up after SARS, MERS and COVID-19: A systematic review and meta-analysis. ERJ Open Res..

[21] Albouaini K., Egred M., Alahmar A., Wright D.J. (2007). Cardiopulmonary exercise testing and its application. Postgrad. Med. J..

